# Preliminary study of neurofilament light chain as a biomarker for hypoxia-induced neuronal injury in dogs

**DOI:** 10.3389/fvets.2023.1284306

**Published:** 2023-10-12

**Authors:** Taesik Yun, Yeon Chae, Yoonhoi Koo, Dohee Lee, Hakhyun Kim, Mhan-Pyo Yang, Byeong-Teck Kang

**Affiliations:** ^1^Laboratory of Veterinary Internal Medicine, College of Veterinary Medicine, Chungbuk National University, Cheongju, Republic of Korea; ^2^College of Veterinary Medicine, Kyungpook National University, Daegu, Republic of Korea

**Keywords:** biomarker, canine, hypoxemia, ischemia, NfL

## Abstract

Neurofilament light chain (NfL) is a neuroaxonal protein in the nervous system. NfL has recently been demonstrated to be a biomarker for various neurological diseases. In this study, we investigated the potential role of NfL in hypoxia-induced neuronal injury in dogs. Serum NfL levels were determined using a single-molecule array. Serum NfL concentrations were significantly higher in hypoxemic dogs without neurological signs (*n* = 6, 175.5 pg/mL) than in healthy dogs (*n* = 15, 15.9 pg/mL; *p* < 0.0001). Therefore, neuronal injury should be considered in dogs with hypoxemia caused by cardiopulmonary diseases, even in the absence of neurological signs.

## 1. Introduction

Hypoxia is a state in which insufficient oxygen reaches the tissues of the body (1). Hypoxia can result from various factors including reduced oxygen tension, hypoventilation, a ventilation-perfusion mismatch, right-to-left shunt, or impaired oxygen diffusion (1). Unlike some other organs, the brain is particularly vulnerable to hypoxia. Neuronal death occurs via apoptosis during cerebral hypoxia (2, 3). Among the numerous causes of hypoxia, cardiopulmonary diseases are the most common in clinical settings, and pulmonary edema and pulmonary hypertension, which are observed in cardiopulmonary diseases, are representative conditions that cause hypoxia in veterinary medicine (4).

Neurofilament light chain (NfL) is a neuroaxonal protein in the nervous system (5–7). NfL has recently been demonstrated to play a role as a biomarker in canine meningoencephalitis of unknown etiology (8, 9). Damaged axons, caused by different etiologies such as degenerative, inflammatory, oncological, traumatic, and vascular diseases, release NfL into the systemic circulation (10).

Hypoxia is common among critically ill veterinary patients. However, it is difficult to determine whether hypoxia-induced neuronal injury occurs in dogs with hypoxemia but without neurological signs. Therefore, this study was performed to determine whether hypoxia-induced neuronal damage occurs and to evaluate whether the serum NfL concentration can serve as a biomarker of hypoxia-induced neuronal damage in hypoxemic dogs without neurological signs.

## 2. Materials and methods

### 2.1. Animals

This retrospective study included six dogs with hypoxemia that visited our hospital between November 2019 and May 2022 as well as 15 healthy dogs whose results were included for comparison. The inclusion criteria for dogs with hypoxemia were as follows: severely hypoxemic dogs whose causes of death were cardiopulmonary diseases (myxomatous mitral valve disease [MMVD], pulmonary hypertension, and bacterial pneumonia) without neurological signs; dogs were defined as severely hypoxemic when they had a peripheral oxygen saturation (SpO_2_) <90% or clinical cyanosis, with SpO_2_ determined using a veterinary pulse oximeter (Oxy9Wave Vet, Bionet, Seoul, South Korea). To minimize errors in pulse oximeter readings due to peripheral circulation impairment, it was ensured that heart rates matched by monitoring electrocardiography for all dogs. Neurological examination was performed for all hypoxemic dogs to determine the absence of neurological signs. Dogs with tumors or heartworms were excluded to avoid the possibility of metastasis to the central nervous system and thromboembolic events, respectively. Dogs with neurological diseases were also excluded. All hypoxemic dogs died of cardiogenic or non-cardiogenic pulmonary edema. The control group consisted of healthy dogs based on blood tests, urinalyses, thoracic and abdominal radiography, and abdominal ultrasound.

### 2.2. Diagnosis of cardiopulmonary diseases

MMVD was diagnosed using echocardiography if the dogs had structural abnormalities (myxomatous degeneration of the mitral valve). Stage C was diagnosed if the dogs had MMVD severe enough to cause signs of heart failure, and stage D was diagnosed if they had clinical signs of heart failure that were refractory to standard treatment (8 mg/kg furosemide daily) (11). The probability of pulmonary hypertension was evaluated based on the peak tricuspid regurgitation velocity and the number of different anatomical sites found on echocardiography (12). A presumptive diagnosis of pneumonia was made based on laboratory data (neutrophilic leukocytosis and a high C-reactive protein concentration), thoracic radiographical findings (abnormal lung patterns), and clinical signs (fever) after excluding other causes (13).

### 2.3. Serum collection and measurement of the NfL concentration

The blood samples for NfL measurement were obtained immediately after the physical examinations including SpO_2_ measurement; they were collected just before death (within a day before death; *n* = 4) due to underlying diseases, or within a day of the occurrence of recurrent congestive heart failure (*n* = 2; MMVD stage D). Blood samples collected from the peripheral or jugular veins were centrifuged at 2000 g for 10 min at room temperature. After centrifugation, the serum was immediately separated and frozen at −80°C until the measurement of NfL concentrations. Serum NfL concentrations were measured as described previously (8, 9). Concentrations were determined using a single-molecule array (Simoa). A Simoa assay kit (Quanterix, Billerica, MA, United States) and a fully automated HD-1 Analyzer (Quanterix, Billerica, MA, United States) were employed. Serum NfL concentrations were measured in duplicate according to the manufacturer’s experimental methods.

### 2.4. Data and statistical analyses

Numerical data are expressed as medians and interquartile ranges. Prism 8 software (GraphPad Software Inc., San Diego, CA, United States) was used for statistical analyses. All tests were two-sided with significance set at *p* < 0.05. The Shapiro–Wilk test was used to determine normal distribution, and normality was not identified. The Mann–Whitney U test was performed to compare the differences in serum NfL concentrations and SpO_2_ between healthy dogs (*n* = 15) and dogs with hypoxemia (*n* = 6).

## 3. Results

### 3.1. Study population

In total, 21 dogs (healthy dogs, *n* = 15; dogs with hypoxemia, *n* = 6) were included in this study. The healthy dogs consisted of three Miniature Poodles, two Pomeranians, two mixed-breed dogs, one Golden Retriever, one Miniature Pinscher, one Spitz, one Shiba Inu, one Bichon Frise, one Yorkshire Terrier, one Maltese, and one Welsh Corgi. The dogs with hypoxemia included three Miniature Poodles, one Maltese, one Pomeranian, and one Cocker Spaniel. The SpO_2_ in the hypoxemic dogs (*n* = 6, 76.5 [75.3–79.3]%) was significantly lower than that in the healthy dogs (*n* = 15, 97 [97–98]%, *p* < 0.0001). Table 1 shows the characteristics of the dogs included in this study.

**Table 1:** Characteristics of dogs in the present study.

		Healthy dogs (*n* = 15)	Hypoxemic dogs without neurological signs (*n* = 6)
Age (years)		5.00 (4.63─8.50)	12.2 (11.6─13.7)
Body weight (kg)		5.14 (4.07─8.53)	3.20 (3.05─4.71)
Sex (number [%])	Male	4 (26.7%)	3 (50.0%)
	Female	11 (73.3%)	3 (50.0%)
The lowest SpO_2_ at the time of sampling (%)		97 (97─98)	76.5 (75.3─79.3)* (ID-1, 80; ID-2, 75; ID-3, 77; ID-4, 85; ID-5, 76; ID-6, 75)
Diagnosis		─	ID-1, MMVD; ID-2, Pneumonia; ID-3, MMVD; ID-4, MMVD, Pneumonia; ID-5, MMVD, PH; ID-6, MMVD, PH
Other data	SBP [mmHg]	─	ID-1, 110; ID-2, 150; ID-3, 150; ID-4, 102; ID-5, 125; ID-6, 140
	CRP [mg/L]		ID-1, 164.3; ID-2, 95.5; ID-3, 115.9; ID-4, 189.2; ID-5, 6.4; ID-6, ─
	WBC [×10^9^ cells/L]		ID-1, 13.0; ID-2, 12.3; ID-3, 29.8; ID-4, 11.9; ID-5, 10.3; ID-6, 12.3
	NT–proBNP[pmol/L]		ID-1, >2000; ID-2, ─; ID-3, >2000; ID-4, >2000; ID-5, >2000; ID-6, >2000

The ages, body weights, and SpO_2_ are expressed as medians and interquartile ranges. Reference intervals: SBP, 90–140 mmHg; CRP, <10 mg/L; WBC, 5.05–16.67 × 10^9^/L; NT–proBNP, <900 pmol/L.

^*^This value was significantly different from that of healthy dogs (*p* < 0.0001). Mann–Whitney U test.

CRP, C–reactive protein; MMVD, myxomatous mitral valve disease; NT–proBNP, N–terminal pro B–type natriuretic peptide; PH, pulmonary hypertension; SBP, systolic blood pressure; WBC, white blood cell.

### 3.2. Nfl concentrations in healthy dogs and dogs with hypoxemia

**Figure 1:**
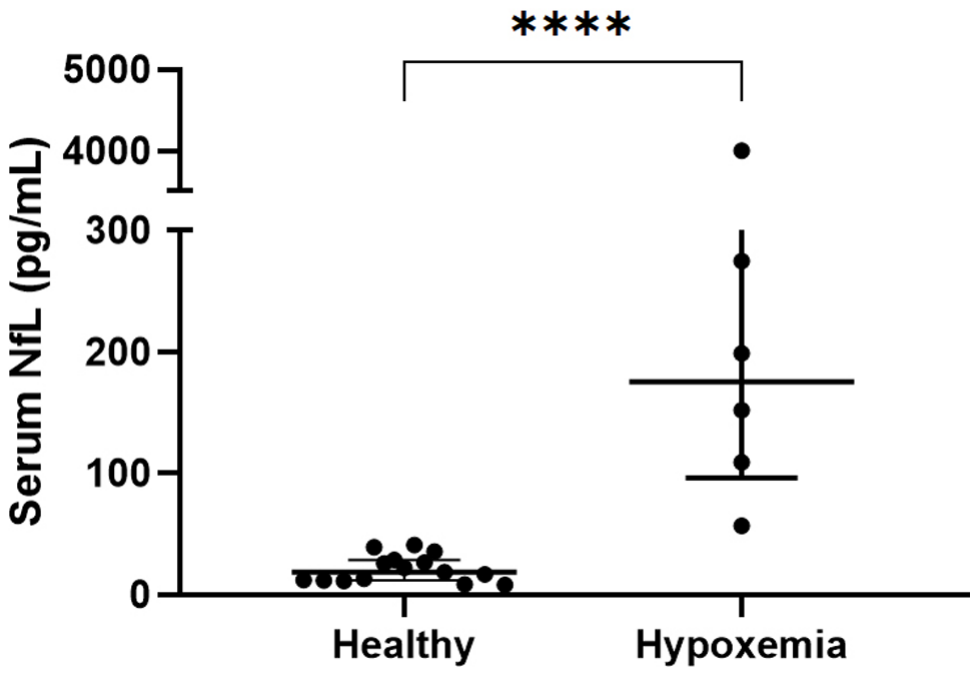
Comparison of serum NfL levels in healthy dogs and hypoxemic dogs without neurological signs. The serum NfL levels differed significantly between the healthy dogs (*n* = 15) and dogs with hypoxemia (*n* = 6); Mann–Whitney U test; ****p < 0.0001; NfL, neurofilament light chain.

The serum NfL concentrations in the hypoxemic dogs (*n* = 6, 175.5 [119.8–256.0] pg/mL) were significantly higher than those in the healthy dogs (*n* = 15, 15.9 [11.7–30.0] pg/mL, *p* < 0.0001, Figure 1).

## 4. Discussion

Cerebral hypoxia develops when the brain does not receive sufficient oxygen. Cerebral hypoxia induces neuronal damage and cellular dysfunction via apoptosis, putting patients in a critical state (2, 3). The representative causes of hypoxia or hypoxemia in veterinary medicine are cardiopulmonary diseases, such as MMVD, pulmonary hypertension, and pneumonia (4). In the present study, it was found that serum NfL, a biomarker of neuroaxonal injury, was elevated in hypoxemic dogs without any neurological signs. Therefore, serum NfL levels can be used as biomarkers to evaluate hypoxic damage to the nervous system.

Hypoxia occurs when an organ receives insufficient oxygen to meet its metabolic needs (1). The brain uses a large amount of energy for its size and weight. Therefore, it has a high metabolic rate and is susceptible to hypoxia. Because the lungs have trouble absorbing oxygen when they have edema or parenchymal abnormalities, any condition that affects them can cause respiratory distress. The major causes of cardiopulmonary arrest are described as the 5Hs and 5Ts, and one of the 5Hs is hypoxia. The 5Hs are as follows: hypovolemia or hemorrhage, hydrogen ions (acidosis), hypoxia or hypoventilation, hyperkalemia or hypokalemia, and hypoglycemia and the 5Ts: thromboembolism or thrombosis, tamponade, toxins, tension pneumothorax, and trauma (14). Therefore, the rapid detection and resolution of hypoxia are important because it can have significant effects on life, including the nervous system.

When cerebral hypoxia occurs, the tissue energy demand cannot be met. Therefore, the adenosine triphosphate (ATP) concentration is reduced, and the loss of ATP leads to the dysfunction of ion pumps, including sodium-potassium pumps (15). When there is ion pump dysfunction, transmembrane ion gradients change, causing membrane depolarization and the opening of voltage-gated ion channels (15). If this hypoxic effect continues, it eventually results in cell death (15). NfL is a promising biomarker for brain injury because it is exclusively expressed in the nervous system and not in other organs (7, 10). Serum NfL concentrations can be used to diagnose and monitor therapeutic responses (clinical signs and lesion size) in humans and dogs (8–10). Therefore, for the prevention and/or treatment of brain injuries, the determination of quantitative biomarkers, such as biochemical parameters (e.g., NfL) of brain damage, to diagnose subclinical lesions at phases when conventional monitoring tools are unable to detect them, could be extremely helpful.

This study had some limitations. First, the primary limitation of this study is the absence of histopathologic evaluation to identify the hypoxia-induced neuronal injury. Second, the NfL levels may vary depending on age, height, and weight (16, 17), but in this study, the signalment of the control group did not match that of the hypoxemic group. Third, it would have been a better comparison if dogs with cardiopulmonary disorders without hypoxemia were included in the control group. Therefore, further studies are needed to complement the limitations mentioned above.

This preliminary study showed that hypoxemic dogs without neurological signs had significantly higher serum NfL concentrations than healthy dogs, immediately before death or within a day of the occurrence of recurrent congestive heart failure. Therefore, in dogs with hypoxemia caused by underlying cardiopulmonary diseases, if elevated NfL levels are verified without neurologic signs, more aggressive treatment of underlying diseases and oxygen supplementation should be provided.

## Data availability statement

The raw data supporting the conclusions of this article will be made available by the authors, without undue reservation.

## Ethics statement

Ethical approval was not required for the studies involving animals in accordance with the local legislation and institutional requirements because this study was conducted retrospectively. Written informed consent was obtained from the owners for the participation of their animals in this study.

## Author contributions

TY: Conceptualization, Data curation, Formal analysis, Investigation, Methodology, Software, Validation, Visualization, Writing – original draft. YC: Writing – original draft, Formal analysis, Software, Visualization, Writing – review & editing. YK: Formal analysis, Investigation, Software, Visualization, Writing – review & editing. DL: Formal analysis, Software, Validation, Visualization, Writing – review & editing. HK: Supervision, Writing – review & editing. M-PY: Supervision, Writing – review & editing. B-TK: Conceptualization, Funding acquisition, Methodology, Project administration, Resources, Supervision, Writing – review & editing.
